# Soy-Based Multiple Amino Acid Oral Supplementation Increases the Anti-Sarcoma Effect of Cyclophosphamide

**DOI:** 10.3390/nu8040192

**Published:** 2016-04-01

**Authors:** Chien-An Yao, Chin-Chu Chen, Nai-Phog Wang, Chiang-Ting Chien

**Affiliations:** 1 Department of Life Science, No. 88, Sec. 4, Tingzhou Road, National Taiwan Normal University, Taipei 11677, Taiwan; yaoc1589@ntuh.gov.tw; 2 Department of Family Medicine, National Taiwan University Hospital, Taipei 100, Taiwan; 3 Biotechnology Center, Grape King Inc., Chung-Li 320, Taiwan; nchinchu@gmail.com; 4 Department of Orthopedic, Kuang-Tien General Hospital, Taichung 433, Taiwan

**Keywords:** apoptosis, autophagy, Atg5, soy-based amino acids, chemotherapy, mice

## Abstract

The use of a mixture of amino acids caused a selective apoptosis induction against a variety of tumor cell lines, reduced the adverse effects of anti-cancer drugs and increased the sensitivity of tumor cells to chemotherapeutic agents. We evaluated the effects and underlying mechanisms of soy-derived multiple amino acids’ oral supplementation on the therapeutic efficacy of low-dose cyclophosphamide (CTX) and on tumor growth, apoptosis, and autophagy in severe combined immunodeficiency (SCID) mice that were injected with sarcoma-180 (S-180) cells. 3-methyladenine or siRNA knockdown of Atg5 was used to evaluate its effect on sarcoma growth. A comparison of mice with implanted sarcoma cells, CTX, and oral saline and mice with implanted sarcoma cells, CTX, and an oral soy-derived multiple amino acid supplement indicated that the soy-derived multiple amino acid supplement significantly decreased overall sarcoma growth, increased the Bax/Bcl-2 ratio, caspase 3 expression, and apoptosis, and depressed LC3 II-mediated autophagy. Treatment with 3-methyladenine or Atg5 siRNA elicited similar responses as CTX plus soy-derived multiple amino acid in downregulating autophagy and upregulating apoptosis. A low dose of CTX combined with an oral soy-derived multiple amino acid supplement had a potent anti-tumor effect mediated through downregulation of autophagy and upregulation of apoptosis.

## 1. Introduction

The main anti-tumor therapies are surgery, radiotherapy, immunotherapy, and chemotherapy. Although the synthetic anti-neoplastic agents used for chemotherapy have potent effects, they can also cause severe adverse effects and lead to multiple drug resistance. Thus, numerous researchers have proposed the use of additional anti-cancer agents such as nutritional supplements and Chinese herbal medicines [1,2,3]. For example, curcumin [4] and sphingomyelin [5] reduced chemotherapy- and radiotherapy-induced side effects. Curcumin given with cisplatin ameliorated fibrosarcoma in animal studies [6,7]. A specific nutrient supplement containing lysine, proline, arginine, ascorbic acid, and green tea extract ameliorated the progression of *N*-methyl-*N*-nitrosourea-induced mammary tumors [8]. Kulcsár *et al.* [9] demonstrated that a mixture of amino acids and other substances (including l-arginine, l-histidine, l-methionine, l-phenylalanine, l-tyrosine, l-tryptophan, l-ascorbate, d-biotin, pyridoxine, riboflavin, adenine, l-malate) had a selective *in vitro* toxic effect against a variety of tumor cell lines, and that this active mixture selectively induced the apoptosis of cancer cells *in vitro*. In cancer patients, the use of dietary nutritional supplements can reduce the adverse effects of anti-cancer drugs [10] and increase the sensitivity of tumor cells to chemotherapeutic agents [4].

The mechanism(s) by which dietary nutritional supplements improve the efficacy of chemotherapy are uncertain. Many cancer therapies kill cancer cells by activating Bax/Bcl-2/caspase or 3/PARP signaling, which increase cell death due to apoptosis, necrosis, autophagy, or pyroptosis [11,12]. Earlier reports indicated an important link between promotion of apoptosis and tumor suppression [13], with the discovery of p53 and proapoptotic *BAX* protein. Autophagy is initially induced to prolong cell survival due to sequestration of cytoplasmic contents into autophagosomes and movement to lysosomes for degradation [14]. Apoptosis and autophagy may be co-regulated in the same directions, and the anti-apoptotic Bcl-2 and Bcl-xL proteins negatively regulate autophagy by binding to Beclin-1 (mammalian Atg6), and apoptosis can suppress autophagy by upregulating the proapoptotic protein Bax and enhancing caspase-mediated cleavage of Beclin-1 [14]. An autophagic survival response occurs in breast cancer cells following nutrient (amino acid) starvation and delayed DNA damage-mediated apoptosis [15]. Thus, when tumor cells are starved from nutrients, oxygen, and blood flow, Beclin-1-mediated autophagy stops cancer cells from dying due to inhibition of apoptosis. Nutritional supplements may mediate a “cross-talk” between apoptosis and autophagy and thereby promote or inhibit tumor progression, although this has not yet been demonstrated.

Microbial fermentation is a rapid, inexpensive, and high-yield production process. In a medium comprised of soymilk and yeast extract, GKB-Aid 1995 overnight cell growth was 10^9^ cfu/mL in a 20-ton bioreactor. About 40% of the fermented product consisted of multiple amino acids (MAA) (Table 1). We hypothesized that the fermented product of GKB-Aid 1995 cells, when prepared with fermented soymilk, may have antioxidant and anti-carcinogenic effects. Therefore, we directly examined the effect of the MAA formula on anti-tumor activity by use of *in vitro* and *in vivo* experiments.

## 2. Experimental Sections

### 2.1. MAA Produced by Fermentation of GKB-Aid 1995 Cells

GKB-Aid 1995 cells were characterized morphologically by Gram’s stain, biochemically by oxidase and catalase tests with a Vitek 2 GN card (BioMerieux, Marcy l’Etoile, France), and genetically by DNA sequencing of the 16s rRNA gene (AppliedBiosystem, Foster City, CA, USA), according to each manufacturer’s instructions. For genotoxicity and acute toxicity tests, GKB-Aid 1995 cells on trypsin soy agar were transferred to a 2.0 L Erlenmeyer flask with 1.0 L broth (composed of 2% sucrose, 1% peptone, 1% yeast extract, and soy milk) and cultured at 32 °C for 24 h on a rotary shaker (120 rpm) for seed culturing prior to a scale-up production step. The scale-up of the fermentation process was performed using the same media in a 20-ton fermenter agitated at 60 rpm with an aeration rate of 0.5 vvm at 32 °C for 24 h. At the end of cultivation, cells in the fermentation medium were heated to 60 °C, lyophilized, reduced to a fine dried powder using a 60 mesh screen, and stored in a desiccator at room temperature.

The resulting GKB-Aid 1995 cells (Bioengineering Center of Grape King Bio Ltd., Chung-Li City, Taiwan) were inoculated on tryptic soy broth agar (1.7% casein peptone, 0.3% soya peptone, 0.5% sodium chloride, 0.25% dipotassium phosphate, 0.25% dextrose, 1.5% agar, pH 7.0) and incubated at 30 °C for 2 days. A single colony was inoculated into a flask with 1.0 L growth medium (4% soybean milk, 1.0% sucrose, 1.0% yeast extract, 1.0% peptone, pH 6.9) at 32 °C on a rotary shaker for 20 h. Then, the 1.0 L flask was added into a 200-L fermentor (Bio Top, Taichung City, Taiwan) agitated at 80 rpm with an aeration rate of 0.5 vvm at 32 °C. The fermentation product was heated at 70 °C for 1 h and stored with aseptic filling in 180 mL bottles. This product contained 17 kinds of amino acids (Table 1).

### 2.2. Animal Care

A total of 160 Male CB17/SCID mice (6–8 weeks old) were used. The animals were housed under pathogen-free conditions at the Center of Laboratory Animal Center, National Taiwan Normal University, at a constant temperature and with light from 700 to 1800 h. Food and water were provided *ad libitum*. We injected mouse sarcoma-180 (S-180) cells to determine the *in vivo* effects of MAA and cyclophosphamide because these cells consistently form rapid tumors in nude mice. All surgical and experimental procedures were approved by Institutional Animal Care and Use Committee of National Taiwan Normal University and were in accordance with the guidelines of the National Science Council of the Republic of China (NSC 1997). All efforts were made to minimize animal suffering and the number of animals used.

**Figure 1:**
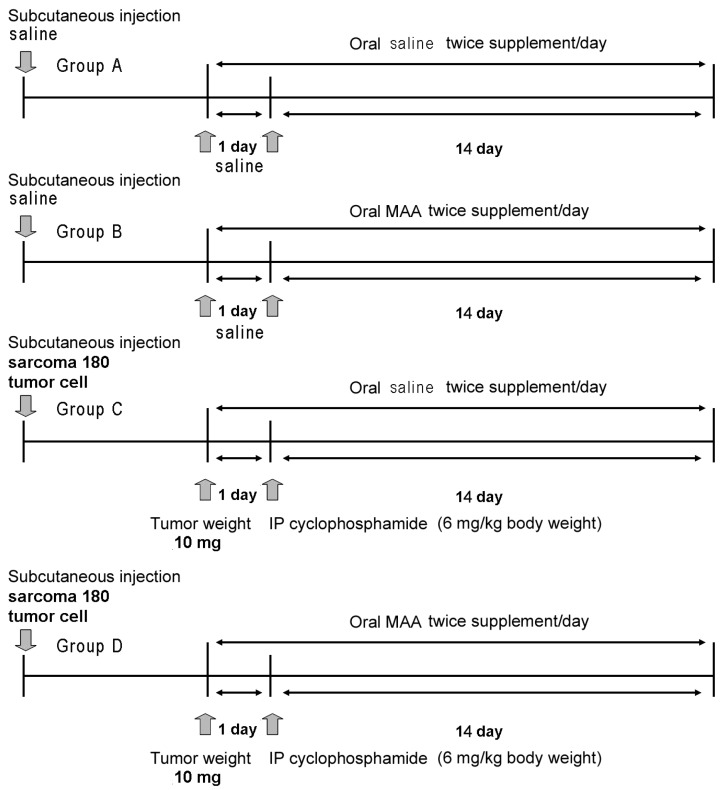
Experimental protocols used to test the effect of a multiple amino acid (MAA) supplement on sarcoma-180 (S-180) bearing mice. Group A, subcutaneous saline followed by oral saline; Group B, subcutaneous saline followed by oral MAA; Group C, subcutaneous S-180 cells followed by cyclophosphamide and oral saline; Group D, subcutaneous S-180 cells followed by cyclophosphamide and oral MAA.

We divided the animals into 4 major groups (A–D), with 40 animals per group (Figure 1). Group A received subcutaneous saline and 4 different doses of oral saline alone (0 mL/20 g, 0.03 mL/20 g, 0.06 mL/20 g, or 0.12 mL/20 g; *n* = 10 in each treatment). Group B received subcutaneous saline and 4 different doses of oral MAA in saline (0×, 0.5× (0.03 mL/20 g), 1× (0.06 mL/20 g), or 2× (0.12 mL/20 g) (*n* = 10 in each treatment). Group C received subcutaneous S-180 cells, intraperitoneal cyclophosphamide monohydrate (6 mg/kg body weight), and 4 different doses of oral saline alone (*n* = 10 in each treatment as above). Group D received subcutaneous S-180 cells, cyclophosphamide monohydrate (6 mg/kg body weight), and 4 different doses of MAA in saline (*n* = 10 in each treatment as above).

### 2.3. Tumors and Treatments

Murine S-180 cells (provided from the Research and Development of Laboratory Animal Center of the National Taiwan University College of Medicine) were grown in a monolayer culture containing humidified air with 5% CO_2_ at 37 °C in RPMI-1640 medium (Sigma, St. Louis, MO, USA) supplemented with 10% fetal calf serum, 100 U/mL penicillin, and 100 mg/L streptomycin. These cells were introduced by subcutaneous injection as previously described [16]. Briefly, the S-180 cells were diluted with sterilized saline at to a concentration of 2 × 10^6^ cells/500 μL, and then inoculated subcutaneously into the right groin region. The Mitutoyo Digimatic caliper was used to measure the tumor in two dimensions, and the volume (m^3^) was calculated using the formula for a prolate ellipsoid (length × width^2^/2). Because the tumors were not removed from the animals, tumor volume was converted to weight by assuming a density of 1.0 g/cm^3^. Tumor volume was evaluated every two days and body weight was measured every week. Animals that received tumor cell injections (Groups C and D) were allocated so that the mean tumor weights of the groups were not statistically different prior to treatment. When the tumor weights were about 10 mg, all groups were fed as described above twice per day for 2 weeks (Figure 1). In Groups C and D, intraperitoneal cyclophosphamide monohydrate (6 mg/kg body weight) in 0.9% saline was administered once.

### 2.4. Tissue Preparation

After 14 days, each animal was sacrificed with an overdose of anesthetics and the tumor was removed for weighing. One part of the tissue was fixed in neutral buffer formalin and the other was immediately frozen in liquid nitrogen and stored at −80 °C for subsequent Western blotting and biochemical analysis.

### 2.5. Western Blotting for Bcl-2, Bax, Cleaved Caspase 3, and LC3 II

Tumor tissue was homogenized in a radio-immunoprecipitation (RIPA) buffer (1.5 M NaCl, 100 mM Tris-base (pH 8.0), 0.5% deoxycholate, 0.1% sodium dodecyl sulfate (SDS), 0.05% aprotinin, and a proteinase inhibitor cocktail) and protein concentration was determined using the Bradford assay. Each sample was mixed with 4× sample buffer (37.5% Tris-HCl, 9% SDS, 0.15% bromophenol blue, and 30% glycerol) and boiled for 10 min. Then, the samples were separated by 12% SDS-PAGE (1.5 M Tris (pH 8.8), 30% acrylamide mix, and 10% SDS, 10% Ammonium persulfate, TEMED) and transferred to nitrocellulose membranes (Amersham Biosciences, Amersham, UK). After blocking with 5% nonfat milk for 1 h, membranes were washed with Tween-20 buffered saline (TTBS) three times for 10 min and incubated overnight at 4 °C with Bcl-2 (1:2000 dilution in TTBS), Bax (1:2000 dilution in TTBS), and cleaved caspase 3 (1:1000 dilution in TTBS), or LC3 II (1:2000 dilution in TTBS), each of which was from Cell Signaling Technology. β-actin was used as the loading control. After washing three times in TTBS, membranes were incubated with secondary antibodies that were conjugated to Bcl-2, Bax, or LC3 II (diluted 1:4000 in TTBS), or conjugated to cleaved caspase 3 (diluted 1:2000 in TTBS), and incubated for 1 h at room temperature. The membranes were again washed with TTBS three times. Immune complexes were visualized with an enhanced chemiluminescence reagent (Amersham). Results were quantified by use of a densitometry using an image analyzing system (Alpha Innotech, San Leandro, CA, USA).

### 2.6. Immunohistochemistry of LC3 II in Tumors

Formalin-fixed tumor segments were cut into 4 μm cross sections, deparaffinized, rehydrated, and blocked by incubation in 3% H_2_O_2_ for 10 min. The sections were incubated in blocking buffer (phosphate-buffered saline, PBS) with 5% BSA (Sigma, St. Louis, MO, USA) for 30 min at room temperature and washed with PBS three times (5 min each). Tissue sections were then incubated with rabbit anti-LC3 II (diluted 1:500 in PBS, Cell Signaling Technology), incubated overnight at 4 °C, then washed with PBS three times (5 min each). The secondary antibodies (Super SensitiveTMNon-Biotin polymer HRP IHC) were used for detection (BioGenex, San Ramon, CA, USA), and then the sections were washed with PBS three times (5 min each). The signal was visualized by incubation with liquid diaminobenzidine tetrahydrochloride. Hematoxylin staining was used to counterstain sections.

### 2.7. TUNEL Staining

TUNEL staining was used to measure DNA fragmentation in deparaffinized, fixed tumor sections according to the manufacturer’s protocol (FragEL DNA Fragmentation kit, Calbiochem, San Diego, CA, USA), and the resulting sections were visualized by fluorescence microscopy. For quantitation, TUNEL-positive nuclei were counted in 5 randomly selected high power (400×) fields, and an average was determined for each section.

### 2.8. Effect of Autophagy Inhibition on CTX-Induced Apoptosis in S-180 Cells

To inhibit autophagosome formation, 3-methyladenine (3-MA) or siRNA knockdown of Atg5 was used to prevent Atg5 expression in S-180 cells. Small interfering RNA for targeting *Atg5* (siAtg5) was from Invitrogen Life Technologies (Carlsbad, CA Santa Cruz Biotechnology) and a universal control siRNA was from Qiagen. The siAtg5 was transfected at a dose of 10 nM with the TransMessenger transfection reagent (Qiagen) according to the manufacturer’s instructions, with 3-MA (10 nM) in a volume of 10 μL PBS. Briefly, a total of 10^6^ cells were plated in 6-cm dishes and transfected using 10 nM siAtg5 and 10 μL of DharmaFECT 1 per dish, with 3-MA or 20 µL of MAA in the normal culture medium using overnight co-treatment with CTX. Following 24 h of incubation with CTX, cells were harvested for Western blotting. All experiments were repeated three times.

### 2.9. Effect of Autophagy Inhibition on CTX-Induced Mitochondrial Leakage of Cytochrome C

Leakage of mitochondrial cytochrome C into the cytosol triggers the mitochondrial apoptotic pathway [17]. Thus, S-180 cells were subjected to differential centrifugation to obtain mitochondrial and cytosolic fractions and protein concentrations were determined with a BioRad Protein Assay (BioRad Laboratories, Hercules, CA, USA). Then, 10 μg of cytochrome C protein (1:1000; Santa Cruz Biotechnology, Inc., Santa Cruz, CA, USA) was electrophoresed in the mitochondrial and cytosolic fractions of sarcoma cells subjected to several treatments.

### 2.10. Statistical Analysis

All values are expressed as means ± standard errors of the means. For comparisons of groups, two-way analysis of variance followed by post-hoc comparisons was used. A *p*-value less than 0.05 was considered to indicate statistical significance.

## 3. Results

### 3.1. Low Dose CTX Combination of Oral MAA Decreased Tumor Growth

**Figure 2:**
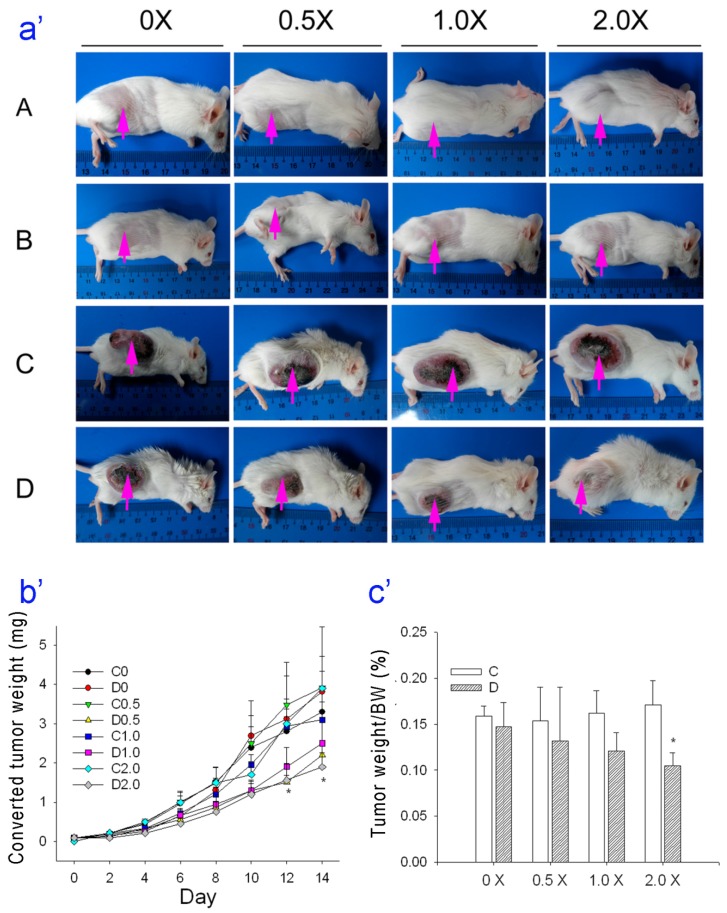
(**a**) Tumor growth in representative mice from each of the four groups (A to D) that were given different amounts of saline (0× to 2×, groups A and C) or different amounts of MAA (0× to 2×, groups B and D); (**b**,**c**) Effect of MAA dosage on tumor growth in mice with S-180 tumors that were given cyclophosphamide + oral saline (C) or cyclophosphamide + oral MAA (D). *n* = 10 in each group. * *p* < 0.05 for a comparison of the D group with the corresponding C group.

Figure 2a shows representative mice from each of the 4 groups (Group A: no tumors with different doses of oral saline; Group B: no tumors with different doses of oral MAA in saline; Group C: tumors, different doses of oral saline, and CTX; and group D: tumors, different doses of oral MAA in saline, and CTX) and Figure 2b,c shows quantitation of these results. As expected, mice in Groups A and B did not develop tumors. For mice with tumors that were given CTX, tumor growth was significantly less for mice given oral MAA (Group D) than for mice given oral saline, and this effect was dose-dependent in terms of tumor weight (D1.0× and D2.0× groups) and tumor weight/body weight (D2.0× group).

### 3.2. Low Dose CTX Combination of Oral MAA Decreased Tumor Growth through Bax Upregulation, Bcl-2 Downregulation, LC3 II Downregulation, Beclin-1 Downregulation and Mitochondrial Injury

**Figure 3:**
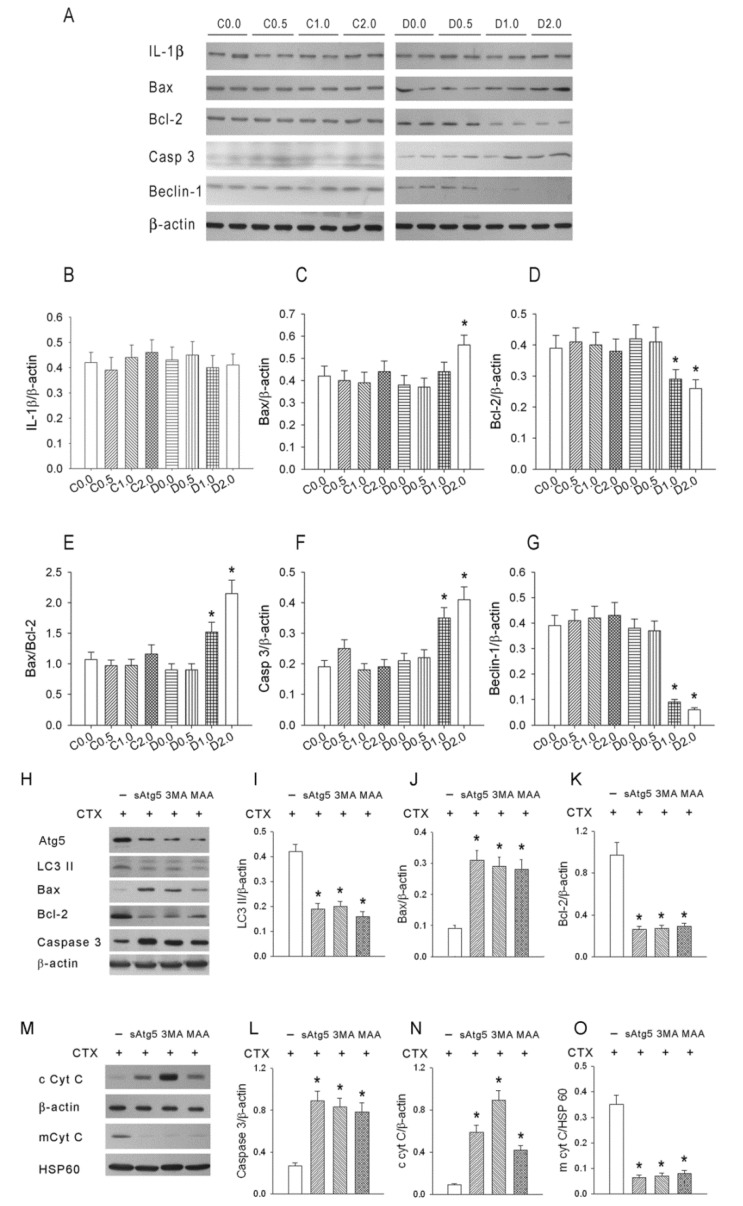
Effect of MAA dosage on molecular biomarkers of three types of cell death in mice with S-180 tumors that were given cyclophosphamide + oral saline (C) or cyclophosphamide + oral MAA (D). (**A**) Representative Western blotting data of IL-1β (pyroptosis mediated protein), Bax, Bcl-2, Casp 3 (apoptosis related proteins), and Beclin-1 (autophagy-related protein); (**B**–**G**) Quantitation of the western blotting results above; (**H**) Representative western blotting data of Atg5, LC3 II, Bax, Bcl-2, and caspase 3 from S-180 cells treated with siRNA Atg5 (siAtg5), 3-MA, or MAA; (**I**–**L**) Quantitation of the Western blotting results above; (**M**) Representative data of cytosolic and mitochondrial cytochrome-c from S-180 cells treated with siRNA Atg5 (siAtg5), 3-MA, or MAA; (**N**–**O**) Quantitation of the Western blotting results above. *n* = 6 in each group. * *p* < 0.05 for a comparison of the D group with the corresponding C group.

Figure 3A shows representative Western blotting results for IL-1β (pyroptosis-related protein), Bax, Bcl-2, Caspase 3 (apoptosis-related proteins), and Beclin-1 (autophagy-related protein) in Group C (tumors, oral saline, CTX) and Group D (tumors, oral MAA, CTX) and Figure 3B–G show quantitation of these results. These figures show no significant differences in IL-1β expression, upregulation of Bax in the D2.0 group, downregulation of Bcl-2 in the D1.0 and D2.0 groups, upregulation of Bax relative to Bcl-2 in the D1.0 and D2.0 groups, upregulation of caspase 3 in the D1.0 and D2.0 groups, and downregulation of Beclin-1 in the D1.0 and D2.0 groups. Taken together, these results indicate that MAA had a significant effect on multiple pathways involved in cell death.

Figure 3H shows representative results of Western blotting experiments for Atg5, LC3 II, Bax, Bcl-2, and caspase 3 in CTX-treated S-180 cells (-) and CTX-treated S-180 cells given different treatments (siAtg5, 3-MA, or MAA) and Figure 3I–L shows quantitation of these results. siAtg5, 3MA, or MAA treatment significantly depressed Atg5 protein expression to 43% ± 5%, 40% ± 4% or 35% ± 4%, respectively. In addition, these results show that each treatment significantly depressed expression of LC3 II and Bcl-2, and increased expression of Bax and caspase 3. Each of these agents also significantly increased leakage of mitochondrial cytochrome C into the cytosol (Figure 3M–O). Taken together, these results show that mitochondrial injury contributes to S-180 cell apoptosis following CTX treatment.

### 3.3. MAA Increased CTX-Induced Tumor Apoptosis in a Dose-Dependent Manner

**Figure 4:**
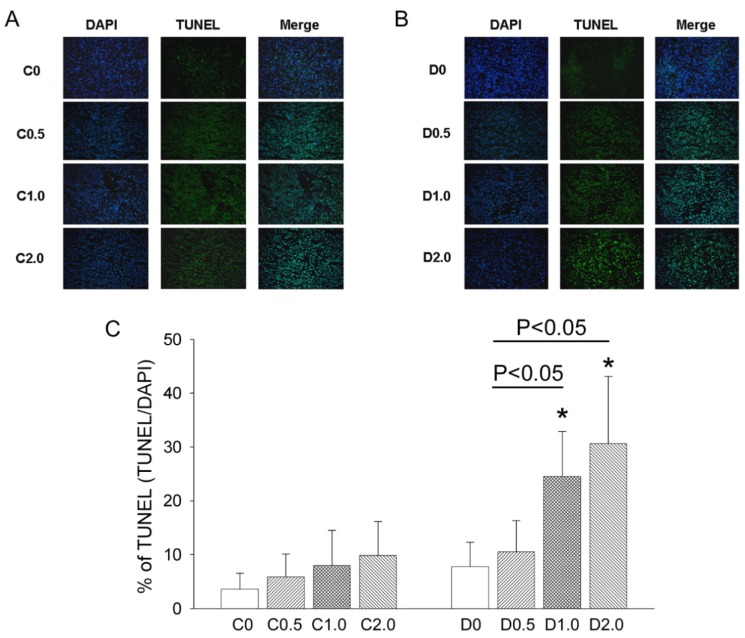
Effect of MAA dosage on apoptosis of sarcoma cells in mice with S-180 tumors that were given cyclophosphamide + saline (C) or cyclophosphamide + MAA (D), with apoptosis determined by the TUNEL assay. (**A**,**B**) Representative staining results (×100) for mice from groups C and D; (**C**) Quantitation of the results above. *n* = 5 in each group. * *p* < 0.05 for a comparison of the D group with the corresponding C group.

Figure 4A,B shows representative results of the TUNEL assay experiments in Groups C and D, and Figure 4C shows quantitation of these results. The results show that significantly more cells were undergoing apoptosis in the D1.0 and D2.0 groups than in the D0 group or in any of the C groups. Thus, MAA increased CTX-induced tumor apoptosis in a dose-dependent manner.

### 3.4. MAA Had a Dose-Dependent Effect on Tumor Cell Autophagy (LC3 II Expression)

**Figure 5:**
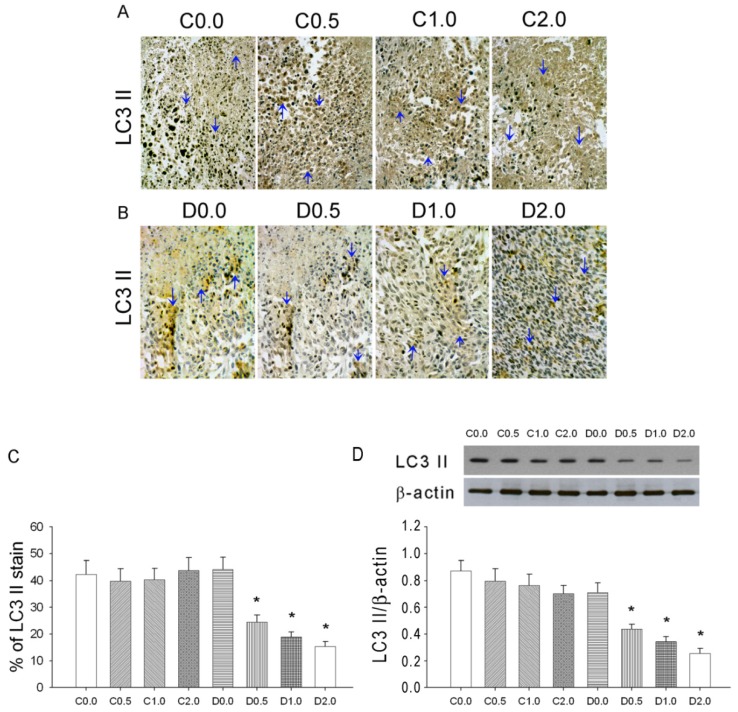
Effect of MAA dosage on LC3 II expression in sarcoma cells of mice with S-180 tumors that were given cyclophosphamide + saline (C) or cyclophosphamide + MAA (D). (**A**,**B**) Representative immunohistochemistry results (×400), with LC3 II-positive cells in brown; (**C**) Quantitation of the results above; (**D**) Representative LC3 II Western blotting graph and quantitation of the results. *n* = 5 in each group. * *p* < 0.05 for a comparison of the D group with the corresponding C group.

Figure 5A,B shows representative figures of LC3 II cell staining (autophagy) of S180 tissues in mice from group C and group D and Figure 5C shows quantitation of these results. These results show that MAA had a dose-dependent effect on tumor cell autophagy (LC3 II expression).

## 4. Discussion

Sarcomas are rare malignant mesenchymal tumors for which there are limited treatment options. In the present study, we evaluated the effect of low-dose CTX combined with an oral MAA supplement on a well-established sarcoma model in mice. Our results indicated that low-dose CTX did not effectively inhibit tumor growth. However, low-dose CTX with oral MAA significantly depressed tumor growth, and this was accompanied by an increased Bax/Bcl-2 ratio, increased caspase 3 expression, and increased apoptosis. CTX plus MAA also significantly depressed LC3 II-mediated autophagy, and the effect of MAA was similar to that of an autophagy inhibitor (3-methyladenine) and Atg5 siRNA in downregulation of LC3 II (autophagy) and upregulation of mitochondrial cytochrome c leakage into the cytosol (apoptosis).

Bcl-2-family proteins regulate all major types of cell death: apoptosis, necrosis, and autophagy. Cells may undergo a highly regulated autophagy to prolong survival, but unregulated autophagy can lead to cell death. Bcl-2 and Bcl-xL suppress autophagy by binding the protein Beclin-1 (ATG7) [12], an essential component of the mammalian autophagy system, which marks autophagic vesicles for fusion with lysosomes, followed by digestion and recycling of components. Autophagy is a vacuolar degradative pathway that is rapidly up-regulated during amino-acid deprivation and is also associated with neurodegenerative diseases, cancer, pathogen infections, and myopathies [5,18]. Autophagy has become an important topic in cancer research, and several recent reviews have examined the role of autophagy in cancer and other diseases [18]. Many anticancer agents can induce autophagy, leading to the suggestion that autophagic cell death may be an important mechanism of tumor cell killing by these agents [19]. For example, a dose- and time-dependent induction of autophagy occurs in tumor cells following cisplatin treatment, as demonstrated by up-regulation of autophagy-inducing protein Beclin-1 and the subsequent development of acridine orange-stained acidic autophagic vesicles [8].

An accurate monitoring of tumor growth (or regression) by a direct mechanical approach using a caliper is critical to determine the effectiveness of an anti-cancer treatment. Three types of cell death—autophagy, apoptosis, and pyroptosis—could potentially contribute to tumor growth or regression in our murine model of sarcoma. In mice given low-dose CTX alone, there were no significant changes in autophagy, apoptosis, and pyroptosis, and sarcoma growth continued. However, in mice given CTX plus MAA (D1.0 and D2.0 groups), there was increased Bax expression, decreased Bcl-2 and Bcl-1 expression, increased caspase-3 expression, and slower tumor growth, but no alteration in IL-1β expression. These results indicate that the MAA supplementation altered autophagy and apoptosis, but not pyroptosis.

As a tumor grows, cancer cells in poorly vascularized areas may undergo autophagy to survive the nutrient-limiting and low-oxygen conditions [5] or as a protection against ionizing radiation [20]. This process may remove damaged macromolecules or organelles, such as mitochondria. We suggest that when tumor cells are starved from nutrients and oxygen (supplied by blood flow), Beclin-1 mediated-autophagy stops cancer cells from dying by inhibiting apoptosis. Autophagy is activated during amino-acid deprivation and is associated with several cancers (5, 18). In our model, tumor growth was much greater in animals given low-dose CTX alone than in mice given CTX plus an oral MAA supplement. The reduced sarcoma growth in the CTX + MAA group was accompanied by enhancement of Bax/Bcl-2/Caspase 3 mediated apoptosis, and inhibition of Beclin-1/LC-3 II mediated autophagy.

Cell homeostasis involves a delicate balance of proliferation, growth arrest, differentiation, apoptosis, and autophagy. Dysregulation of apoptosis frequently accompanies cancer pathogenesis. Diverse nutrient supplements or deprivations of substrates such as amino acids, selenium, and the cruciferous vegetable constituent phenethyl isothiocyanate [21,22,23], may promote apoptosis and tumor regression. In an *in vivo* tumor xenograft model, the combination of a leucine-free diet and an autophagy inhibitor synergistically suppressed the growth of human melanoma tumors and triggered widespread apoptosis of cancer cells [21]. Our study was consistent with these findings that anticancer effects can be obtained by combining inhibition of autophagy and enhancement of apoptosis.

There is evidence that inhibition of autophagy can induce apoptosis in human hepatoma cell line HepG2 [24]. In our study, autophagy occurred in cultured sarcoma cells and was downregulated by MAA, 3-methyladenine, and Atg5 siRNA. Our data showed that sarcoma cell apoptosis increased after CTX + MAA treatment. MAA, 3-methyladenine, and Atg5 siRNA inhibited autophagy, decreased LC3 II expression, and increased cell apoptosis. There was also increased expression of Bax, release of cytochrome c into the cytosol, cleavage of caspase-3, and increased apoptosis in the sarcoma. Apoptosis is typically accompanied by increased production of reactive oxygen species (ROS) [11]. Thus, MAA may have enhanced the anti-cancer activity of CTX and Atg5 siRNA may have mediated the downregulation of autophagy due to ROS generation and activation of the mitochondrial apoptosis pathway. Hypoxia is common in solid tumors, and leads to cancer cell chemoresistance by provoking adaptive responses including autophagy. Wu *et al.* [25] demonstrated that hypoxia significantly protected cancer cells from cisplatin-induced cell death in a HIF-1α- and HIF-2α-dependent manner. Augmented induction of autophagy by hypoxia decreased lung cancer cell susceptibility to cisplatin-induced apoptosis and inhibited autophagy by 3-MA or siRNA targeted ATG5 effectively attenuated cisplatin resistance under hypoxia [25]. Autophagy, a lysosomal degradation pathway, is inhibited by the interaction of cellular Bcl-2 with a key autophagy effector, Beclin-1 [26]. Kulcsár *et al.* [9] hypothesized that the small molecules that selectively accumulate in cancer cells might participate in a non-immunological antitumor surveillance mechanism.

We found that treatment of tumor-bearing mice with an MAA oral supplement inhibited the growth of sarcomas by 40%–69%. In agreement, previous research indicated that a mixture of amino acids with 5-fluorouracil or cisplatin led to enhanced tumor inhibition through the mitochondrial apoptosis pathway and to G1 arrest in cancer cells [9]. Moreover, Besirli *et al.* [27] found that activation of autophagy in injured photoreceptor cells inhibited fas-mediated apoptosis. These findings suggest that use of an MAA supplement in combination with other anti-tumor agents as a novel approach for treatment of cancer. Our data using MAA plus CTX also indicated that the MAA supplement mixture enhanced the anti-tumor effect of CTX by inhibiting autophagy and enhancing apoptosis in the sarcoma cells.

In summary, a dose of 6 mg/kg CTX did not effectively inhibit tumor growth under our experimental conditions. However, treatment of mice with CTX and an oral MAA supplement suppressed tumor growth and increased the apoptosis of tumor cells. The underlying mechanism appears to be a decrease of Bcl-2, an increase of Bax, an increased ratio of Bax/Bcl-2, and an increase of caspase 3. We also found that autophagy (Beclin-1 expression) decreased in mice given CTX + MAA relative to those given CTX + saline. Taken together, our results demonstrated that a low dose of CTX combined with an oral MAA supplement inhibited tumor growth by enhancement of apoptosis and inhibition of autophagy.

## Figures and Tables

**Table 1:** Composition of the soy-based multiple amino acid (MAA) supplement.

MAA	Hydrolyzed Amino Acid Composition	Percentage
1	Aspartic acid (Asp)	4.85%
2	Threonine (Thr)	1.91%
3	Serine (Ser)	1.63%
4	Glutamic Acid (Glu)	8.26%
5	Proline (Pro)	1.98%
6	Glycine (Gly)	2.11%
7	Alanine (Ala)	2.66%
8	Cystine (Cys)	0.15%
9	Valine (Val)	2.12%
10	Methionine (Met)	0.70%
11	Isoleucine (Ile)	1.50%
12	Leucine (Leu)	3.00%
13	Tyrosine (Tyr)	1.23%
14	Phenylalanine (Phe)	1.65%
15	Histidine (His)	0.87%
16	Lysine (Lys)	2.30%
17	Arginine (Arg)	2.40%
	Total	39.32%

## Abbreviations

CTX	cyclophosphamide
3-MA	3-methyladenine
MAA	multiple amino acids
PBS	phosphate-buffered saline
RIPA	radio-immunoprecipitation
S-180	sarcoma-180
SCID	severe combined immunodeficiency
SDS	sodium dodecyl sulfate
siAtg5	small interfering RNA for targeting Atg5
TTBS	Tween-20 buffered saline
