# Correction: Yao, C.A., et al. Soy-Based Multiple Amino Acid Oral Supplementation Increases the Anti-Sarcoma Effect of Cyclophosphamide. *Nutrients* 2016, *8,* 192

**DOI:** 10.3390/nu12092732

**Published:** 2020-09-08

**Authors:** Chien-An Yao, Chin-Chu Chen, Nai-Phog Wang, Chiang-Ting Chien

**Affiliations:** 1 Department of Life Science, No. 88, Sec. 4, Tingzhou Road, National Taiwan Normal University, Taipei 11677, Taiwan; yaoc1589@ntuh.gov.tw; 2 Department of Family Medicine, National Taiwan University Hospital, Taipei 100, Taiwan; 3 Biotechnology Center, Grape King Inc., Chung-Li 320, Taiwan; nchinchu@gmail.com; 4 Department of Orthopedic, Kuang-Tien General Hospital, Taichung 433, Taiwan

The authors wish to make the following correction to their published paper [1]. The second from the left panel (D 0.5) in Figure 5B is overlayed with the leftmost image (D 0.0). See original figure below. 

The correct Figure 5B should be as below: 
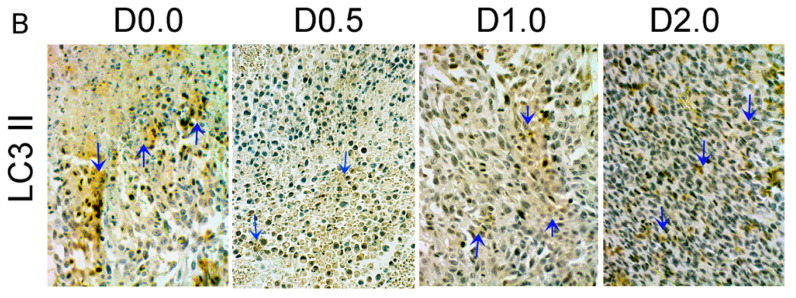


The authors would like to apologize to the readers of *Nutrients* for this mistake. The change made does not affect the results. The published version will be updated on the article webpage with a reference to this Correction.

## Figures and Tables

**Figure 5:**
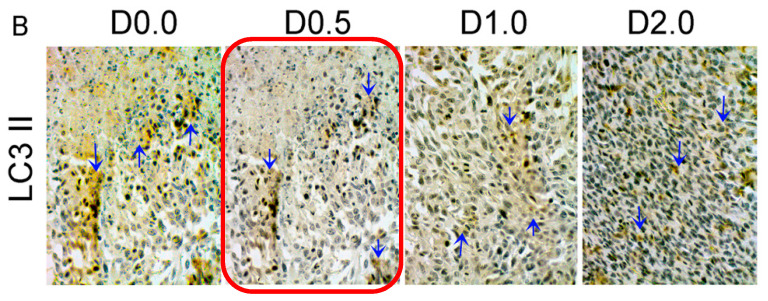
**(original).** Incorrect image D 0.5 in the published version. The image in the red box (D 0.5) was merged with the image D 0.0.
